# Angiotensin-(1-7) Central Mechanisms After ICV Infusion in Hypertensive Transgenic (mRen2)27 Rats

**DOI:** 10.3389/fnins.2021.624249

**Published:** 2021-04-23

**Authors:** Lucas M. Kangussu, Marcella Nunes Melo-Braga, Bruna Soares de Souza Lima, Robson A. S. Santos, Hélida Monteiro de Andrade, Maria José Campagnole-Santos

**Affiliations:** ^1^National Institute of Science and Technology in Nanobiopharmaceutics (INCT-Nanobiofar), Federal University of Minas Gerais, Belo Horizonte, Brazil; ^2^Department of Morphology, Federal University of Minas Gerais, Belo Horizonte, Brazil; ^3^Department of Biochemistry and Immunology, Federal University of Minas Gerais, Belo Horizonte, Brazil; ^4^Department of Parasitology, Federal University of Minas Gerais, Belo Horizonte, Brazil; ^5^Department of Physiology and Biophysics, Institute of Biological Sciences, Federal University of Minas Gerais, Belo Horizonte, Brazil

**Keywords:** angiotensin-(1-7), hypothalamus, hypertensive transgenic (mRen2)27 rats, cytokines, iNOS, ROS modulators

## Abstract

Previous data showed hypertensive rats subjected to chronic intracerebroventricular (ICV) infusion of angiotensin-(1-7) presented attenuation of arterial hypertension, improvement of baroreflex sensitivity, restoration of cardiac autonomic balance and a shift of cardiac renin-angiotensin system (RAS) balance toward Ang-(1-7)/Mas receptor. In the present study, we investigated putative central mechanisms related to the antihypertensive effect induced by ICV Ang-(1-7), including inflammatory mediators and the expression/activity of the RAS components in hypertensive rats. Furthermore, we performed a proteomic analysis to evaluate differentially regulated proteins in the hypothalamus of these animals. For this, Sprague Dawley (SD) and transgenic (mRen2)27 hypertensive rats (TG) were subjected to 14 days of ICV infusion with Ang-(1-7) (200 ng/h) or 0.9% sterile saline (0.5 μl/h) through osmotic mini-pumps. We observed that Ang-(1-7) treatment modulated inflammatory cytokines by decreasing TNF-α levels while increasing the anti-inflammatory IL-10. Moreover, we showed a reduction in ACE activity and gene expression of AT1 receptor and iNOS. Finally, our proteomic evaluation suggested an anti-inflammatory mechanism of Ang-(1-7) toward the ROS modulators Uchl1 and Prdx1.

## Graphical Abstract

Hypothalamic mechanisms induced by ICV infusion of angiotensin-(1-7) in the transgenic (mRen2)27 hypertensive animals. Ang-(1-7) treatment modulated components of the renin-angiotensin system (RAS), attenuating increased levels of AT1 and ACE activity, and negatively modulated inflammatory profile, by increasing anti-inflammatory IL-10 and decreasing TNFα and iNOS in the hypothalamus. Further, altered oxidative stress/inflammation by ROS mediator, decreasing Uchl1 (ubiquitin carboxyl-terminal hydrolase isozyme L1) while increasing antioxidant peroxiredoxin 1 (figure was created with pictures from biorender.com).

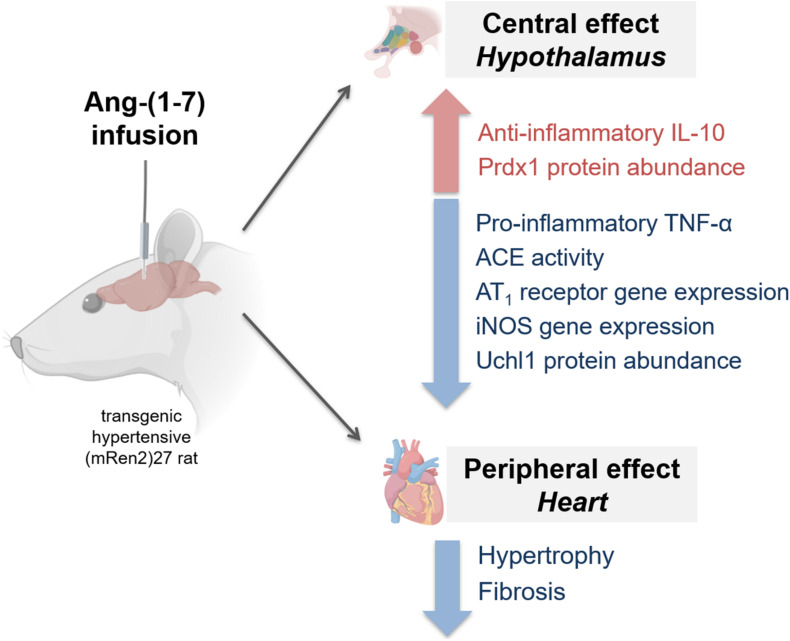

## Introduction

Angiotensin-(1-7) [Ang-(1-7)] is a key component of the renin-angiotensin system (RAS) and an important modulator of cardiovascular function (Santos et al., 2018). Its actions mainly counterbalance those effects of Angiotensin II (Ang II) in different tissues and several pathophysiological conditions (Santos et al., 2018). Of note, it is important to highlight that Ang-(1-7) actions go beyond modulation of the cardiovascular system and include effects on inflammation, stress coping behaviors, and neurodegenerative diseases (Kangussu et al., 2013;

Rodrigues-Machado et al., 2013; Almeida-Santos et al., 2017; Magalhaes et al., 2018; Santos et al., 2018).

We have shown Ang-(1-7), upon Intracerebroventricular (ICV) infusion, improves baroreflex control of heart rate (HR), especially its bradycardic component, both in normotensive or hypertensive animals (Campagnole-Santos et al., 1992; Oliveira et al., 1996; Britto et al., 1997; Heringer-Walther et al., 2001; Guimaraes et al., 2012). Additionally, ICV infusion of Ang-(1-7) inhibited sympathetic and increased vagal drive to periphery in rabbits with chronic heart failure, thus contributing to improve baroreflex gain also in this condition (Kar et al., 2011). In addition, ICV infusion of Ang-(1-7) for 4 weeks significantly reduces the expression of Ang II and AT1 receptors in the brain of spontaneously hypertensive rats (Jiang et al., 2013). Besides, ICV administration of Ang-(1-7) (Dobruch et al., 2003) or the delivery of an Ang-(1-7) fusion protein in the cisterna magna (Garcia-Espinosa et al., 2012) attenuated high blood pressure of the hypertensive (mRen2)27 rats. Of note, the actions of Ang-(1-7) in the brain seem to be mostly mediated by Mas receptor (Kangussu et al., 2015; Santos et al., 2018), which was shown to be expressed in different areas (Becker et al., 2007; Freund et al., 2012).

Findings from our laboratory showed that ICV infusion of Ang-(1-7) attenuated hypertension, normalized baroreflex control of arterial pressure and the autonomic tone to the heart, and also prevented the increase in cardiac collagen type I mRNA expression in DOCA-salt hypertensive rats (Guimaraes et al., 2012). Furthermore, we also showed that Ang-(1-7)/Mas activation in the brain is capable of reducing cardiac hypertrophy and pre-fibrotic lesions and decreasing the altered imbalance of Ang II/Ang-(1-7) in the heart of hypertensive transgenic rats (mRen2)27 (Kangussu et al., 2015). These effects occurred in association with the improvement of baroreflex control of HR and cardiac autonomic control and decreased blood pressure. Although several brain effects were described, the central mechanisms triggered by Ang-(1-7) are still not fully understood.

In the present study, we evaluated whether a chronic increase in Ang-(1-7) in the brain could modulate inflammatory cytokines and expression/activity of RAS components in the hypothalamus of normotensive and hypertensive transgenic rats (mRen2)27. Furthermore, we have performed a proteomic analysis to evaluate the mechanisms involved in the central beneficial effects of Ang-(1-7) in arterial hypertension.

## Materials and Methods

### Animals

Male heterozygous (mRen2)27 rats (TG; 10 to 12 weeks old) and age-matched normotensive control Sprague-Dawley (SD) rats were obtained from the animal facility of the Laboratory of Hypertension, Institute of Biological Sciences, Federal University of Minas Gerais (UFMG), Brazil. Rats were housed in the animal facility and kept at controlled room temperature (22–24°C) and 12/12 h light/dark cycle. (mRen2)27 transgenic rat, that overexpress a mouse renin gene, is an interesting model of experimental hypertension, in which kidney and plasma renin activity are suppressed (Mullins et al., 1990) and the development and maintenance of hypertension has been attributed to the high activity of renin, and perhaps angiotensin II, in extrarenal tissues, such as, the adrenal glands, heart, brain and blood vessels (Bader et al., 1992; Campbell et al., 1995).

All procedures used in this study were approved and strictly followed the ethical principles of animal experimentation adopted by the Ethics Committee on Animal Use of Federal University of Minas Gerais and institutionally approved under protocol number 49/2013.

### Chronic Intracerebroventricular (ICV) Infusion

Rats were anesthetized with tribromoethanol (25 mg/100 g of b.w., i.p.), for ICV infusion, a metallic cannula (guide cannula) was implanted into the right lateral ventricle (from the bregma: AP −1.0 mm; LL 1.5 mm; and DV −4.5 mm and cemented with three anchoring screws to the skull). ICV cannula was connected via vinyl tubing to an osmotic mini-pump (ALZET, model 2004), which was implanted subcutaneously between the scapulae. The infusion rate was 0.5 μl/h for 14 days. After surgery, rats received a poly-antibiotic (20U; Pentabiotico^®^, Fort Dodge, Brazil) and flunixin meglumine (1 mg/Kg, s.c.; Banamine^®^, Schering Plough, Brazil) for post-operation analgesia. The control groups normotensive (SD saline or SD CT) and transgenic hypertensive rats (mRen2)27 (TG saline or TG CT) received sterile isotonic saline. SD A7 and TG A7 groups received Ang-(1-7) at 200 ng/h. The site of infusion was verified postmortem by the presence of Alcian blue dye (5%), injected through the ICV cannula (2 μL), only in the ventricular system. Ang-(1-7) was purchased from Bachem, Germany.

### Heart Histological Analysis

In a sub-group of animals, the heart beat was stopped in diastole using KCl (10%, i.v.). The heart was fixed in 10% neutral-buffered formalin solution and stained with hematoxylin and eosin for cell morphometry. Three sections (5 μm; with 10 μm intervals between each section) from each animal were visualized in a light microscope (BX41^®^; Olympus, Center Valley, PA, United States) photographed (Q-Color3^TM^; Olympus, Center Valley, PA, United States) under 400x magnification and analyzed using the ImageJ software. Cardiomyocytes diameters of the left ventricular wall (∼50 cardiomyocytes for each animal) were measured across the region corresponding to the nucleus. Only cardiomyocytes cut longitudinally with nuclei and cellular limits visible were considered for analysis. All analyses were performed in a double-blind way by the same researcher. Levels of hydroxyproline in heart tissue were measured using the hydroxyproline assay kit (Sigma-Aldrich, St. Louis, MO, United States) according to the manufacturer instructions.

### Evaluation of Cytokines in the Brain

Fourteen days After ICV infusion, all groups Had the hypothalamus extracted and homogenized (100 mg/mL of extraction solution). Brain homogenate Was centrifuged at 3,000 × *g* for 10 min at 4°C. the supernatant Was collected and stored at −20°C. Concentrations of interleukin-1α (IL-1α), interleukin-6 (IL-6), tumor necrosis factor-alpha (TNF-α) and interleukin-10 (IL-10) Were measured using commercially available antibodies according to the manufacturer (R&D Systems, Minneapolis, MN, United States) by enzyme-linked immunosorbent assay (ELISA). Results Were expressed as pg/100 mg of tissue.

### Measurement of Angiotensinergic Receptors and Inducible Nitric Oxide Synthase (iNOS) mRNA Expression

Total RNA was obtained following the TRIzol reagent method (Invitrogen, Life Technologies, United States) according to the manufacturer’s protocol. RNA samples (2 μg) were treated with DNase to eliminate genomic DNA present in the samples. mRNA expression was assessed by qRT-PCR after reverse transcription with MML-V (Moloney murine leukemia virus) (Invitrogen Life Technologies, United States). The cDNA for endogenous S26 ribosomal (endogenous control) and AT_1_, AT_2_, Mas receptors and iNOS were amplified using specific primers using SYBR Green reagent (Applied Biosystems, Foster City, NY, United States). The reactions were performed using 40 cycles and annealing temperature of 60°C (ABI Prism 7000, Applied Biosystems, Foster City, NY, United States). Gene expression was quantified using the comparative Ct (threshold cycle) method. Primers sequence of AT_1_: 5′-GGT GGG AAT ATT GGA AAC AG-3′ (forward) and 5′-AAG AAG AAA AGC ACA ATC GCC-3′ (reverse); AT_2_: 5′-GCT GAG TAA GCT GAT TTA TG-3′ (forward) and 5′-TTA AGA CAC AAA GGT GTC CA-3′ (reverse); Mas receptor: 5′-CCC ACC CAT TCC CAT AGT GC-3′ (forward) and 5′-CCG AGA GGA GAG ATG CTC ATG-3′ (reverse); iNOS: 5’-CCT TGT TCA GCT ACG CCT TC-3’ (forward) and 5’-GGT ATG CCC GAG TTC TTT CA-3’ (reverse); endogenous control S26: 5′-CGA TTC CTG ACA ACC TTG CTA TG-3′ (forward) and 5′-CGT GCT TCC CAA GCT CTA TGT-3′ (reverse).

### Measurement of ACE and ACE2 Activity

Hypothalamus ACE activity was measured as previously described (Santos et al., 1985). Briefly, the hypothalamus homogenate was incubated at 37°C with the ACE substrate hippuryl-His-Leu (1 mM; Sigma-Aldrich) in a total volume of 500 μl buffer (0.4 M sodium borate buffer, 0.3 M NaCl, pH 8.3) in the presence or absence of the ACE-specific inhibitor captopril (10 μM), for 30 min. Following incubation, 120 μl of 0.3 N NaOH and 10 μl o-phthaldialdehyde (20 mg/ml in methanol) were added. After 10 min at room temperature, 20 μl of 3 N HCl was added and the tubes were centrifuged at 16,000 × *g* in a tabletop microcentrifuge for 5 min. Supernatants were transferred to black 96-well microplate. Fluorescence (excitation wavelength of 355 nm, emission wavelength of 485 nm) was measured using a FLUOstar Optima plate reader (BMG Labtechnologies, Durham, NC, United States). The rate of substrate cleavage was determined by comparison with a standard curve of the His-Leu product.

The enzymatic activity of ACE2 was determined using a fluorogenic substrate (fluorogenic peptide VI; R&D Systems, United States). Enzymatic activity was measured with a Spectra Max Gemini EM Fluorescence Reader (Molecular Devices, United States), as previously described (Huang et al., 2003; Huentelman et al., 2004). Samples were read every minute for 60 min, beginning immediately after adding the fluorogenic peptide substrate at 37°C. The result of each sample was expressed as arbitrary units (a.u.) corresponding to the average of the fluorescence measured in the maximum velocity of the reaction, corrected for mg of protein measured by the Bradford method (Bradford, 1976).

### Proteomic Analysis Based on Two-Dimensional Gel Electrophoresis (2DE)

Proteomic analysis was performed in hypothalamus from independent biological replicates of each group, SD CT (*n* = 4), SD A7 (*n* = 5), TG CT (*n* = 5), and TG A7 (*n* = 6). The first step was the removal of fat from the sample by washes with chloroform/methanol/water (4:8:3) v/v, for 1 h under stirring followed by centrifugation at 1,500 × *g* for 10 min at room temperature (Folch et al., 1957; Bligh and Dyer, 1959). This washing procedure was repeated three times. After this, the sample was allowed to dry at 4°C for 16 h. Then, to extract protein, the hypothalamus was resuspended in lysis buffer (8M urea, 2M thiourea, 4% CHAPS, 65 mM DTT, 40 mM Tris base, and a protease inhibitor mix (GE Healthcare, United States). The sample was incubated under agitation for 2 h and then centrifuged at 10,000 × *g* for 30 min. The soluble fraction was obtained and maintained at −80°C until use. According to the manufacturer’s instructions, protein content was measured using the 2D-Quant kit (GE Healthcare, United States).

The experiment was conducted as described by Lima et al. (2017). Briefly, we first analyzed all independent biological replicates from each group with Coomassie 2D gels. We confirmed a high reproducibility (coefficient of variation ≤10%) regarding the total number of spots, their relative positions, and intensities (data not shown). Next, we performed the DIGE analysis with 150 μg of a pool containing biological replicates of each four animal groups labeled with 400 pmol fluorophore CyDye^TM^ (Cy2, Cy3, and Cy5) (GE Healthcare, United States), according to the experimental design (Table 1). A mixture of protein extracts from the four groups was labeled with Cy2 as an internal standard. The experiments were performed in triplicate. Then, the samples were loaded onto IPG strips (18 cm, pH 4–7; GE Healthcare, United States) for overnight rehydration at room temperature, following to IEF on an Ettan IPGphor system (GE Healthcare, United States) at 20°C and a maximum current of 50 μA/strip (see more detail in Lima et al., 2017). After reduction and alkylation, the strips were applied to a 12% SDS-PAGE within low-fluorescence glass plates (GE Healthcare, United States). The 2D-gel electrophoresis was performed in the dark using an Ettan DALT 6 unit (GE Healthcare, United States). Gels images were obtained on a Typhoon Trio laser imager (GE Healthcare, United States) and analyzed using DeCyder 2D software, Version 7.0 (GE Healthcare, United States). The statistic *t*-test with false discovery rate correction was used with α < 0.05 of significance. Protein spots that showed high abundance in each animal group (*p*-value < 0.01) or qualitatively (detected in only one condition in a specific comparison) were selected for MS identification. The DIGE gels were also stained with colloidal CBB G-250 following procedures previously described (Neuhoff et al., 1988) to improve manually excised of selected spots. Spots were processed as described by Costa et al. (2011). Tryptic peptides were analyzed with a MALDI-ToF-ToF AB Sciex 5800 (AB Sciex, Foster City, CA, United States) mass spectrometer, following protein search using MASCOT as previously described (Lima et al., 2017).

**TABLE 1:** DIGE experimental design.

**Gel**	**Cy3**	**Cy5**	**Cy2**
1	TG A7	TG CT	Internal standard
2	SD A7	SD CT	
3	SD A7	TG A7	
4	TG CT	SD A7	
5	TG A7	SD CT	
6	SD CT	TG CT	

*Schematic Cy Dye labeling for each gel that contains a specific pool of protein extract from hypothalamus for each group.*

### Statistical Analysis

Data are expressed as mean ± SEM. Differences among groups were assessed by one-way ANOVA followed by Newman-Keuls *post hoc* test and were performed with GraphPad Prism software (version 6.0). All values were expressed as mean ± standard error of the mean (SEM). Statistical significance was assumed for all values at *p* < 0.05.

## Results

### Chronic Ang-(1-7) ICV Infusion Decreased Cardiac Hypertrophy in TG Rats

**FIGURE 1:**
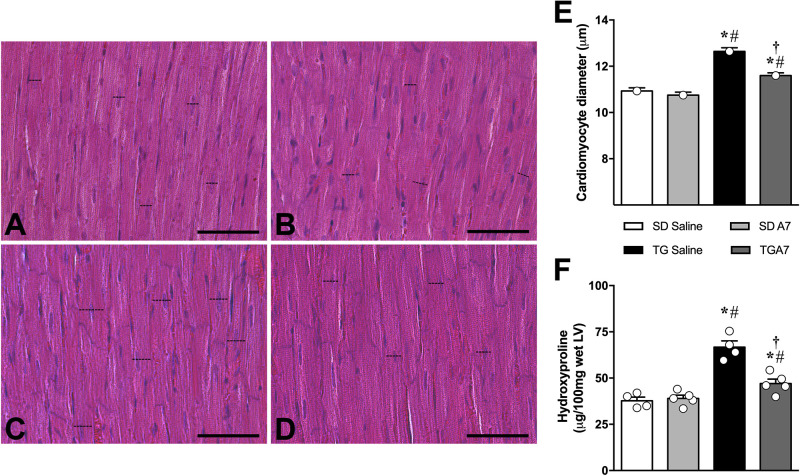
Representative photomicrographs of sections of the left ventricle of sections control (SD saline, *n* = 5); **(A)**, control treated with Ang-(1-7) (SD A7, *n* = 4); **(B)**, hypertensive rats control (TG saline, *n* = 4); **(C)** and hypertensive rats treated with ICV Ang-(1-7) (TG A7, *n* = 5); **(D)**. Bar = 20 μm. Stain: hematoxylin/eosin. The cardiomyocyte diameter **(E)** of the free wall LV and Hydroxyproline as a fibrotic marker **(F)**. *p < 0.05 vs. SD saline; ^#^*p* < 0.05 vs. SD A7; ^†^*p* < 0.05 vs. TG saline (One-way ANOVA followed by Newman-Keuls *post hoc* test).

In order to confirm our previous result, we have first evaluated alterations on cardiac structures, hypertrophy and fibrosis, in hypertensive rats subjected to chronic Ang-(1-7) ICV infusion by histology. Figure 1A–D shows representative images of the histology of the different groups. As expected, morphometric analysis of the images showed that TG animals presented cardiomyocytes hypertrophy (12.6 ± 0.16 μm, *n* = 6; Figures 1C,E) when compared to SD group (10.9 ± 0.13 μm, *n* = 6; Figures 1A,E). As we showed in previous work (Guimaraes et al., 2012; Kangussu et al., 2015), hypertensive animals treated with Ang-(1-7) showed significant attenuation of cardiac hypertrophy (11.6 ± 0.11 μm, *n* = 6; Figures 1D,E). Similar results were obtained when the level of hydroxyproline, a biochemical marker of collagen deposition, was evaluated (Figure 1F). TG CT group revealed a remarkable increased hydroxyproline level (66.7 ± 1.3 μg, *n* = 5; Figure 1F) in the LV when compared to SD CT group (37.7 ± 1.9 μg, *n* = 5; Figure 1F) while ICV treatment with Ang- (1-7) attenuated hydroxyproline levels in TG animals (47.0 ± 2.37 μg, *n* = 5; Figure 1F).

### Chronic Ang-(1-7) ICV Infusion Modulated Cytokines in the Hypothalamus of TG Rats

**FIGURE 2:**
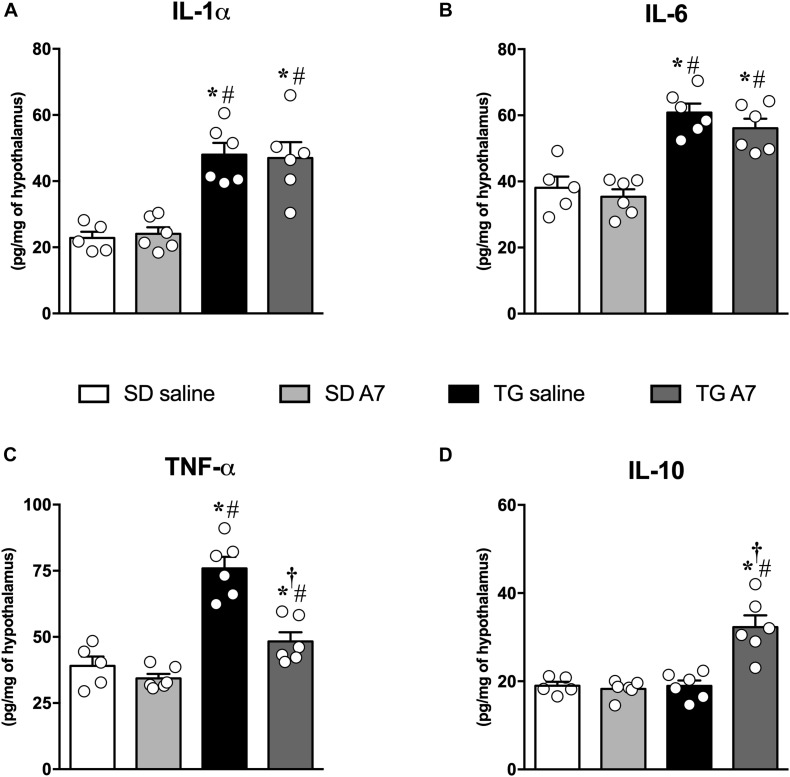
Concentrations of pro-inflammatory cytokines in the hypothalamus in SD and hypertensive rats treated with Ang-(1-7) ICV. interleukin-1 alpha (IL-1α) **(A)**, interleukin-6 (IL-6) **(B)**, tumor necrosis factor-alpha (TNF-α) **(C)** and anti-inflammatory cytokine, interleukin-10 (IL-10) **(D)** were measured using commercially available antibodies by enzyme-linked immunosorbent assay (ELISA). Results are expressed as mean ± SEM. *n* = 5 or 6. *p < 0.05 vs. SD saline; ^#^ *p* < 0.05 vs. SD A7; ^†^*p* < 0.05 vs. TG saline (One-way ANOVA followed by Newman-Keuls *post hoc* test).

One of our hypotheses for the central mechanism associated with the anti-hypertensive effect of Ang-(1-7) in transgenic hypertensive rats was a modulation of inflammatory mediators levels, as cytokines in the hypothalamus. As expected, TG animals showed higher levels of different pro-inflammatory cytokines in comparison to SD control group: IL-1α (48 ± 3.5 vs. 23 ± 1.8 pg/mg – Figure 2A), IL-6 (61 ± 2.7 vs. 38 ± 3.4 pg/mg – Figure 2B) and TNF-α, (76 ± 4.3 pg/mg vs. 39 ± 3.5 pg/mg – Figure 2C). No change was observed for the anti-inflammatory cytokine, IL-10 (Figure 2D). ICV infusion of Ang-(1-7) mitigated TNF-α levels in TG group (48 ± 3.4 pg/mg, *n* = 6; Figure 2C) when compared to untreated TG rats (76 ± 4.3 pg/mg, *n* = 6; Figure 2C). Moreover, ICV Ang-(1-7) significantly increased IL-10 levels in TG rats (32 ± 2.6 pg/mg vs. 19 ± 1.2 pg/mg, untreated TG, *n* = 6 each; Figure 2D). Ang-(1-7) did not change IL-1α and IL-6 levels in the hypothalamus of TG rats (Figures 2A,B, respectively).

### Evaluation of Renin-Angiotensin System (RAS) Components in the Hypothalamus of TG Rats

**FIGURE 3:**
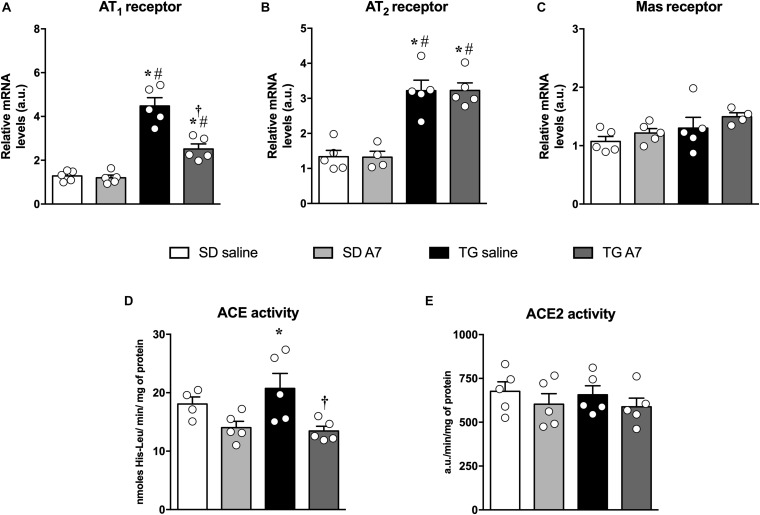
Expression or activity of renin-angiotensin components in the hypothalamus in SD and hypertensive rats treated with Ang-(1-7) ICV. AT_1_ receptor **(A)**, AT_2_ receptor **(B)**, Mas receptor **(C)**, ACE activity **(D)**, and ACE2 activity **(E)**. Results are expressed as mean ± SEM. *n* = 4 or 5. *p < 0.05 vs. SD saline; ^#^*p* < 0.05 vs. SD A7; ^†^*p* < 0.05 vs. TG saline (One-way ANOVA followed by Newman-Keuls *post hoc* test).

Among the main pathophysiological mechanisms of arterial hypertension is the hyperactivity of the renin-angiotensin system (RAS), characterized by an increase in Ang II in plasma and tissue concentration, as previously reported in these hypertensive transgenic rat model (Bader et al., 1992; Campbell et al., 1995; Nakagawa and Sigmund, 2017). Therefore, we evaluated the alteration in gene expression of main angiotensinergic receptors. Hypertensive untreated TG rats showed increased gene expression of AT_1_ (4.5 ± 0.5 a.u.; Figure 3A) and AT_2_ receptor (3.2 ± 0.3 a.u., Figure 3B) when compared to SD group (1.2 ± 0.1 a.u. and 1.3 ± 0.2 a.u., respectively; *n* = 5 each; Figures 3A,B). Interestingly, chronic administration of Ang-(1-7) was able to attenuate AT_1_ receptor mRNA expression in the hypothalamus of hypertensive animals (2.5 ± 0.25 a.u., *n* = 5; Figure 3A) without altering the increased AT_2_ receptor gene expression (3.2 ± 0.2 a.u., *n* = 5; Figure 3B). Gene expression of Mas receptor was not altered in untreated or Ang-(1-7) treated TG rats (Figure 3C).

Regarding the activity of ACE, TG hypertensive untreated rats showed increased activity (21 ± 2.5 nmoles of His-Leu/min/mg of protein, *n* = 5; Figure 3D) when compared to normotensive control animals (17 ± 1.3 nmoles of His-Leu/min/mg of protein; *n* = 4; Figure 3D). Chronic Ang-(1-7) ICV infusion induced a pronounced reduction in ACE activity in the hypothalamus of TG rats to the level of the normotensive control group (13 ± 0.7 nmoles of His-Leu/min/mg of protein, *n* = 5; Figure 3D). In addition, there was no alteration in ACE2 activity in the hypothalamus among all groups (Figure 3E).

### Evaluation of iNOS Expression in the Hypothalamus

**FIGURE 4:**
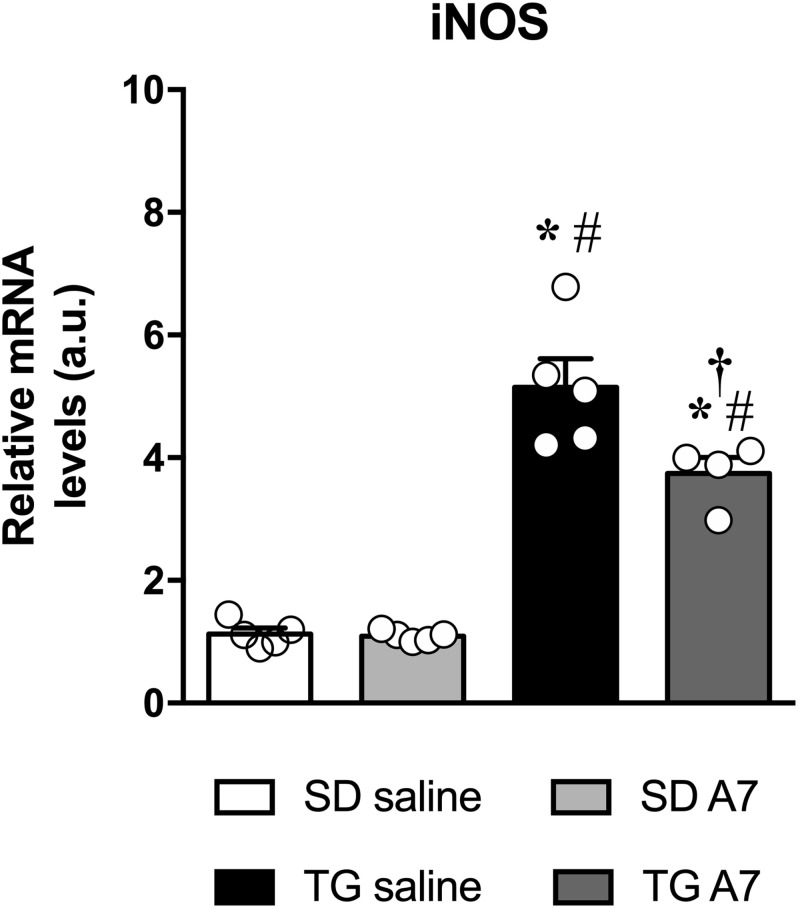
Expression of mRNA for iNOS in the hypothalamus in SD and hypertensive rats treated with Ang-(1-7) ICV. Results are expressed as mean ± SEM. *n* = 4 or 5. *p < 0.05 vs. SD saline; ^#^*p* < 0.05 vs. SD A7; ^†^*p* < 0.05 vs. TG saline (One-way ANOVA followed by Newman-Keuls *post hoc* test).

Another important protein in the pathophysiology of hypertension is iNOS (Oliveira-Paula et al., 2014). Therefore, we have measured iNOS gene expression in the hypothalamus of TG rats. As can be seen in Figure 4, hypertensive untreated TG rats showed increased gene expression of iNOS (5.1 ± 0.4 a.u., *n* = 5) when compared to SD group (1.1 ± 0.1 a.u.; 1.3 ± 0.2 a.u., *n* = 5). Ang-(1-7) ICV was able to attenuate the increased iNOS gene expression in the hypothalamus of TG animals (3.7 ± 0.2 a.u., *n* = 5).

### Proteomic Analysis of Hypothalamus After Chronic ICV Infusion of Ang-(1-7)

**FIGURE 5:**
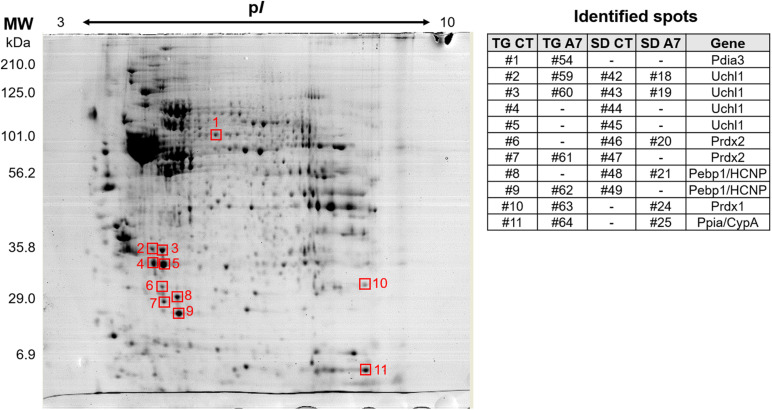
Representative 2-DE gel image of DIGE-stained proteins (gray view). The represented gel is from the TG CT group and the squares indicate the identified spots. The panel on the right shows the number of the matched spots identified in each analyzed group, where (-) means not detected. The panel also include the gene from the identified protein. Pdia3, protein disulfide-isomerase A3; Uchl1, ubiquitin carboxyl-terminal hydrolase isozyme L1; Prdx2, peroxiredoxin-2; Pebp1/HCNP, phosphatidylethanolamine-binding protein 1; Prdx1, peroxiredoxin-1; Ppia/CypA, peptidyl-prolyl *cis-trans* isomerase A/cyclophilin A.

Next, the effect of Ang-(1-7) ICV infusion in transgenic (mRen2)27 hypertensive animals was evaluated at the proteome level by combining 2D electrophoresis and MALDI-TOF/TOF approaches. Figure 5 shows a representative 2-DE gel indicating protein spots, which had matched identification between the groups in the different comparison aimed in this work (TG CT vs. TG A7, SD CT vs. SD A7, and SD CT vs. TG CT). Our results revealed a highly similar profile of all four analyzed conditions (SDCT, SDA7, TGCT and TGA7), containing few qualitative and significant quantitative differences, as shown in Table 2. Treatment with Ang-(1-7) in TG hypertensive rats negatively modulated three proteins (Uchl1, Prdx2 and Pebp1, also known as HCNP) and one proteoform (Uchl1), while positively modulated Prdx1. In the SD animals, ICV Ang-(1-7) positively regulated three proteins (Prdx2, Prdx1, and Ppia, also known as CypA) and negatively regulated one protein, ubiquitin carboxyl-terminal hydrolase isozyme L1 (Uchl1) and its proteoform. Notably, treatment with Ang-(1-7) decreased the expression level of two proteoforms of Uchl1 while increased the abundance of Prdx1 in both normotensive and hypertensive groups. On the other hand, we observed opposing expression levels for Prdx2 in SD and TG groups after chronic infusion of Ang-(1-7), i.e., upregulation in the former and downregulation in the latter group. However, it seems to be two different proteoforms, since they were identified with different mass and pI (Table 2). Moreover, three proteins were differentially regulated in TG CT in comparison to SD CT (Pdia3, Prdx1, and Ppia; Table 2).

**TABLE 2:** Identification of differentially expressed proteins in the hypothalamus of Sprague-Dawley (SD) and TGR (mRen2)27 (TG) rat treated with 14 days of Ang-(1–7) (A7) or saline ICV infusion the control group (CT).

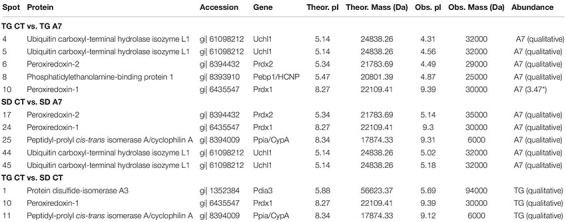

gi, NCBI database accession number; Theor, theoretical; pI, isoelectric point; Obs, observed.

*Fold change TG A7/TG CT (%volume), *p* < 0.01.

*Table shows only the proteins that were differentially changed in the 3 comparisons presented, i.e., TG-A7 vs. TG, SD-A7 vs. SD and TG vs. SD. Qualitative means that using our approach and considering its limit of detection, the specific protein/proteoform was detected (up red arrow) or not detected (down blue arrow) in one of the group of the comparison (A7 or TG).*

## Discussion

Ang-(1-7) mechanisms of action in the heart have been extensively studied and its protective role is well recognized (Santos et al., 2018). We have previously shown that Ang-(1-7) ICV treatment lowered blood pressure and improved cardiac function in hypertensive rats (Guimaraes et al., 2012; Kangussu et al., 2015), as well as attenuated metabolic syndrome induced by fructose intake (Guimaraes et al., 2014). However, the mechanisms triggered by Ang-(1-7) in the brain are yet not very well understood. Here, we present the effect of ICV infusion with Ang-(1-7) on mediators in the hypothalamus of hypertensive transgenic (mRen2)27 rats. These effects include a decrease in TNF-α, decrease in ACE activity, reduction in gene expression of Ang II AT_1_ receptor and iNOS, an increase in the anti-inflammatory cytokine, IL-10, and alteration in proteins related to ROS modulation with decreased abundance of Uchl1 and increased abundance of Prdx1.

It has been shown that neurogenic models of hypertension present increase reactive oxygen species, activation of NF-κB and production of TNF-α in the brain. These effects indicate an activation of glial cells and the production of pro-inflammatory cytokines, thus contributing to the neurohumoral excitation observed in hypertension (Kang et al., 2008; Sriramula et al., 2008). The hypothalamus is one important site in the CNS where inflammatory signals are involved in pathophysiology of hypertension. Studies have shown that main neuroactive cytokines involved in hypothalamic inflammatory mechanisms related to cardiovascular diseases are TNF-α, IL-6, IL1-α/IL-1β, and IL-10. TNF-α and IL-1-β can increase the activity of cyclooxygenase-2 in perivascular macrophages to generate prostaglandin E2, increasing the discharge of PVN neurons, which in turn regulate adrenocorticotropic hormone release, sympathetic outflow, and ultimately blood pressure elevation (Khor and Cai, 2017). Following this research line, Kang et al. (2009) have investigated the involvement of RAS components and brain cytokines in the induction of heart failure by ligation of the anterior descending coronary artery. The authors found that ICV infusion with losartan, SN50 (NF-κB nuclear translocation inhibitor) or tempol (superoxide anion scavengers) significantly attenuated gene expression of AT_1_, NADPH oxidase, NF-κB-p50 receptors, as well as the production of pro-inflammatory cytokines in the PVN. Moreover, Shi et al. (2010) showed that microglial activation and production of pro-inflammatory cytokines in PVN contributes to the neurogenic hypertension pathophysiology. In addition, inhibition of microglial activation (reduced production of pro-inflammatory cytokines) or overexpression of IL-10 (an anti-inflammatory cytokine) in PVN through viral transfection attenuates Ang II-induced hypertension (Shi et al., 2010).

Interesting observations were made by Cardinale et al. (2012), among the pathophysiological mechanisms of Ang II-induced hypertension, an important modulation of NF-κB in PVN. Chronic treatment with a sequence of inhibition of NF-κB bilaterally in PVN for 14 days was able to reduce blood pressure, reduce the activity of the p65 subunit of NF-κB, pro-inflammatory cytokines, reactive oxygen species, AT_1_ receptor expression and ACE activity in PVN (Cardinale et al., 2012). Additionally, Sriramula et al. (2013) demonstrated that the infusion of Ang II for 28 days in Sprague-Dawley rats induced a significant increase in mean arterial pressure and cardiac hypertrophy. Interestingly, hypertension and cardiac hypertrophy were alleviated by ICV administration of Etanercept (TNF-α inhibitor). Infusion of Ang II also induced an increase in the expression of ACE and the AT_1_ receptor and reduced the expression of ACE2 and the Mas and AT_2_ receptors in PVN. Treatment with the TNF-α inhibitor was able to reverse all these changes (Sriramula et al., 2013). Among its central actions, Ang-(1–7) can exert direct effects on microglia lowering the release pro-inflammatory cytokines, as well as, by attenuating the prorenin-induced increases in pro-inflammatory cytokines (Liu et al., 2016). In addition, PVN overexpression of ACE2 attenuates the increase in TNF-α, IL-1β, and IL-6 (Sriramula et al., 2011).

Therefore, taken together, our results are in line with these findings showing that our transgenic hypertensive animals have higher levels of pro-inflammatory cytokines (e.g., TNF-α, IL-1α, and IL-6), higher ACE activity and higher expression of AT_1_ receptor. Further, we now advanced these observations by showing, for the first time, the chronic ICV infusion of Ang-(1-7) in (mRen2)27 rats modulate the RAS components (decreasing AT_1_ receptor and ACE activity) and inflammatory mediators (diminishing TNF and increasing IL-10) in the hypothalamus. It is possible the reduction in ACE activity may also contribute to increase hypothalamic level of Ang-(1-7), since, ACE not only reduce Ang II generation but also decrease Ang-(1-7) degradation. These effects, combined with the reduction of AT_1_ expression, positively contributes to ultimately decrease BP and fibrotic cardiac effect of Ang-(1-7). Although, Ang-(1-7) treatment did not alter the high levels of IL-1α and IL-6 in (mRen2)27 rats, the shift in the hypothalamic inflammatory condition caused by the alteration in the relationship between the important pro- and anti-inflammatory mediators, TNF-α and IL-10, have certainly contributed to lower blood pressure and to mitigate cardiac remodeling in (mRen2)27 rats. Of note, IL-10 is a potent anti-inflammatory and immune regulatory cytokine that contributes importantly to curtailing the inflammatory response, and more importantly to promoting resolution of inflammation. In addition to reduce the level of pro-inflammatory cytokine production by activated CNS cells (Moore et al., 2001), IL-10 can alter microglial phenotype polarization from the predominantly inflammatory “M1” phenotype to a more immunoregulatory “M2” phenotype that expresses protective and/or repairing factors (Deng et al., 2012; Mingomataj and Bakiri, 2016). Thus, IL-10 is generally considered to be the quintessential immunosuppressive cytokine produced within the CNS (Burmeister and Marriott, 2018).

In hypertensive states, elevated levels of reactive oxygen species (ROS) or reactive nitrogen species such as superoxide anion (O_2_^–^) and nitric oxide (NO), respectively, may be altered in medullary areas related to cardiovascular control, such as rostral ventrolateral medulla (RVLM) (Tai et al., 2005), caudal (CVLM) (Braga et al., 2011) and hypothalamic area (Khor and Cai, 2017). While relatively small amounts of NO plays an important role in cardiovascular homeostasis, high NO levels may have detrimental consequences to the cardiovascular system and contribute to hypertension (Oliveira-Paula et al., 2014). NO in the CNS, including the brainstem and hypothalamus, plays an important role in the regulation of blood pressure via the sympathetic nervous system. Its enzymatic formation is derived from three types of NO synthase (NOS): neuronal, endothelial and inducible. The latest, also known as iNOS, is not usually expressed in cells, but its expression can be induced by cytokines, for example, and the exaggerated expression of iNOS can lead to hypertension (Zanzinger, 1999; Patel et al., 2001; Kimura et al., 2005). Kimura et al. (2005, 2009) showed that overexpression of iNOS in the rostral ventrolateral medulla (RVLM) activates the sympathetic nervous system, inducing hypertension, probably by an increase in oxidative stress in this area. Furthermore, iNOS levels in the RVLM were significantly higher in SHR than in Wistar-Kyoto rats (WKY). Furthermore, a decreased BP and heart rate in SHR, but not in WKY, was observed after bilateral microinjection of aminoguanidine (iNOS inhibitor) into the RVLM. Our present results show that, as expected, (mRen2)27 rats presented high levels of iNOS in the hypothalamus. Interestingly, ICV Ang-(1-7) infusion mitigates this increase, consequently attenuating the hypertension effects.

In the present study, we also observed a higher protein abundance of Ppia/CypA in (mRen2)27 hypertensive compared to normotensive rats. Ppia/CypA facilitates protein folding, and under inflammatory conditions, such as oxidative stress, Ppia/CypA can be secreted. Its extracellular form stimulates pro-inflammatory mediators in endothelial cells and vascular smooth muscle cells (VSMC) (Nigro et al., 2013). In fact, others have shown that Ppia/CypA enhanced NF-κB activity by promoting its stability and nuclear translocation (Sun et al., 2014). Besides, Ppia/CypA is also involved in the ROS production, vascular remodeling, cardiac hypertrophy, matrix degradation, and it has been shown that Ang II induces Ppia/CypA release (Satoh et al., 2009; Nigro et al., 2013).

Also comparing hypertensive and normotensive rats, our findings showed an increased level of Ang II AT_2_ receptor gene expression, protein abundance of antioxidant Peroxiredoxin-1 (Prdx1) and protein folding disulfide-isomerase A3 (Pdia3). These results indicate a compensatory fight response mechanism against the higher stress situation on (mRen2)27 rats to preserve cell function and survival. The peroxiredoxins (Prdx) are antioxidant enzymes that protect the organism against hydrogen peroxide (H_2_O_2_)-induced oxidative stress. The balance of the redox system is essential since high levels of ROS induce apoptosis (Finkel, 1998). Therefore, peroxiredoxins have an important role in cellular homeostasis by decreasing H_2_O_2_ levels and consequent downstream responses (Chae et al., 1999). Several molecules induce the production of H_2_O_2_, such as TNF-α and the downstream effects include the expression of pro-inflammatory molecules as IL-1 and IL-6 (Kang et al., 2004). Also, cytosolic peroxiredoxins have been suggested to be an important regulator of TNF signaling pathways (Kang et al., 2004). Therefore, the increase in Pdrx1 expression may counterbalance H_2_O_2_ and consequently reduce the expression of pro-inflammatory effectors such as TNF-α to avoid apoptosis and cell death. However, the redox balance still favors the pro-inflammatory side. The other compensatory mechanism related to higher ROS levels is associated with Pdia3, a chaperon protein involved in reconstructing misfolded proteins by disulfide bond formation. Pdia3 has a neuroprotective role against the aggregation of ROS-induced misfolding proteins (Andreu et al., 2012). A pro-apoptotic function has been related to Pdia3, providing a link between unfolded protein and apoptotic signaling (Zhao et al., 2015).

Our proteomic data also revealed that treatment with Ang-(1-7) decreased the expression level of two proteoforms of the ubiquitin carboxyl-terminal hydrolase isozyme L1 (Uchl1) in both normotensive and hypertensive groups. Uchl1 is highly expressed in the brain and is required for axonal integrity maintenance. It has also been suggested to be a biomarker for traumatic brain injury (Papa et al., 2010). This protein belongs to the ubiquitin system and has a significant role in the regulation of several cellular processes, controlling protein activity and abundance. Misfolding proteins are usually ubiquitinated and then degraded via the 26S proteasome or by lysosomal degradation. The dimer form of Uchl1 has ubiquitin-ligase activity, while its monomer form functions as a deubiquitylating enzyme (Bishop et al., 2016). Moreover, Uchl1 promotes H_2_O_2_ production by upregulating NADPH oxidase 4 (NOX4) activity through deubiquitination in the migration process of cancer cells (Kim et al., 2015), as well as in angiogenesis (Song et al., 2018). Furthermore, Ang-(1-7) treatment increased the levels of the antioxidant Prdx1 in both groups while increased the level of antioxidant peroxiredoxin-2 (Prdx2) only in the SD treated group. As mentioned before, these proteins are important regulators of oxidative stress and inflammation by decreasing the H_2_O_2_ levels and consequent its downstream responses. Therefore, one of the anti-inflammatory mechanisms triggered by Ang-(1-7) seems to be decreasing the levels of ROS, such as H_2_O_2_, by decreasing the abundance of ROS generation modulator Uchl1 while increasing ROS scavengers Prdx1 and Prdx2.

Surprisingly, we found a decreased abundance of the Prdx2 after Ang-(1-7) infusion in the hypertensive group. As mentioned before, this proteoform is different from the other identified with higher abundance after Ang-(1-7) infusion in the normotensive group. Therefore, we believe they may have different functions. Furthermore, the decreased protein level of Prdx2 in TG treated could be an indirect effect of Ang-(1-7) that leads to a reduced level of inflammatory TNF-α and a higher level of anti-inflammatory IL-10, suggesting a lower level of ROS by upstream molecules as the already mentioned Uchl1 and Prdx1. Ang-(1-7) treatment also decreased the expression level of phosphatidylethanolamine-binding protein 1 (Pebp1 or HCNP), also called Raf kinase inhibitor protein (RKIP), in hypertensive animals. Pebp1 is the precursor of hippocampal cholinergic neurostimulating peptides (HCNP) (Ling et al., 2014). RKIP inhibits the transcription factor NF-κB and MAPK signaling independently. It is a regulatory mechanism of NF-κB activation in response to pro-inflammatory cytokines stimulation as TNF-α and IL-1β (Yeung et al., 2001). As mentioned before, the abundance of these cytokines is lower in TG treated group suggesting NF-κB reduced activity. The higher level of Pebp1 in the TG control group may also be a compensatory mechanism to counterbalance the remarkable inflammation observed in these animals.

Finally, we would like to point out some of the limitations of the present study. First, the hypothalamus is intricately involved in the development and maintenance of arterial hypertension. However, it comprises different nuclei and areas when activated were shown to either raise or lower BP, mainly by altering sympathetic nervous activity. The hypothalamic nuclei are closely interconnected and also communicate with many other areas in the central nervous system both rostral and caudally. Thus, future evaluations of the protein profile expressed in each nucleus/region may extend the observations made in the present study, which were not possible at this study due to the amount of total protein in each sample and the method available for proteomic evaluation. Second, the study was carried out only in male rats, however, there are important gender differences in this TG rat related to the RAS, such as, prorenin, Ang I, and Ang II were higher only in transgenic males compared with females (Lee et al., 1996). Thus, it is crucial and perhaps even mandatory, that in future studies gender comparisons are done. Knowing better the effect of treatments or maneuvers on female animals and women will certainly improve the treatment and control of high blood pressure. Third, we have not validate the results of protein expression with Western blotting. Although some debate exists in the literature regarding the value of this type of validation, future studies should evaluate the expression of the specific proteins altered. Furthermore, future studies should also reproduce these protein changes in experimental models to provide more insights on the role of these proteins for the pathophysiology of hypertension.

## Conclusion

We provide evidence that the chronic infusion of Ang-(1-7) in the brain modulates inflammatory mediators, RAS components and iNOS in the hypothalamus, suggesting a possible additional anti-hypertensive mechanism for Ang-(1-7) in the CNS. Moreover, we highlight one of the anti-inflammatory mechanisms of Ang-(1-7) could be by decreasing Uchl1 abundance while increasing Prdx 1 and, subsequently decreasing ROS production in the oxidative stress and inflammation (Graphical abstract). Although proteins evaluation by Western blotting are still required to confirm the proteomic analysis, the data of this study reinforce that pharmacological strategies leading to brain accumulation of Ang-(1-7) may become alternative therapies to treat arterial hypertension, especially those of neurogenic or resistant nature.

## Data Availability Statement

The original contributions presented in the study are included in the article/supplementary material, further inquiries can be directed to the corresponding author/s.

## Ethics Statement

All experimental protocols were approved by the Institutional Committee that regulates the use of laboratory animals – Comitê de Ética no Uso de Animais (CEUA/UFMG, protocol #49/2013) and were conducted in accordance with the National Institutes of Health (NIH) Guide for the Care and Use of Laboratory Animals.

## Author Contributions

LK contributed in conception and design of the research, performed the experiments, analyzed the data, prepared the figures, interpreted the results of experiments, writing, review, and editing the manuscript, and approved the final version of the manuscript. MM-B analyzed the data, prepared the figures, interpreted the results of experiments, writing, review, and editing the manuscript, and approved the final version of the manuscript. BS performed the experiments, analyzed the data, prepared the figures, interpreted the results of experiments, and approved the final version of the manuscript. RS interpreted the results of experiments, reviewed the manuscript, and approved the final version of the manuscript. HA contributed in conception and design of the research, analyzed the data, interpreted the results of experiments, reviewed the manuscript, and approved the final version of the manuscript. MC-S contributed in conception and design of the research, analyzed the data, prepared the figures, interpreted the results of experiments, writing, review, and editing the manuscript, and approved the final version of the manuscript. All authors contributed to the article and approved the submitted version.

## Conflict of Interest

The authors declare that the research was conducted in the absence of any commercial or financial relationships that could be construed as a potential conflict of interest.
